# Distinct Signaling Cascades Elicited by Different Formyl Peptide Receptor 2 (FPR2) Agonists

**DOI:** 10.3390/ijms14047193

**Published:** 2013-04-02

**Authors:** Fabio Cattaneo, Melania Parisi, Rosario Ammendola

**Affiliations:** Department of Molecular Medicine and Medical Biotechnology, University of Naples Federico II, Via S. Pansini 5, 80131 Naples, Italy; E-Mails: fabio.cattaneo@unina.it (F.C.); parisi_me@libero.it (M.P.)

**Keywords:** FPR2, signal transduction, nicotinamide adenine dinucleotide phosphate (NADPH) oxidase, cell signaling, transactivation

## Abstract

The formyl peptide receptor 2 (FPR2) is a remarkably versatile transmembrane protein belonging to the G-protein coupled receptor (GPCR) family. FPR2 is activated by an array of ligands, which include structurally unrelated lipids and peptide/proteins agonists, resulting in different intracellular responses in a ligand-specific fashion. In addition to the anti-inflammatory lipid, lipoxin A4, several other endogenous agonists also bind FPR2, including serum amyloid A, glucocorticoid-induced annexin 1, urokinase and its receptor, suggesting that the activation of FPR2 may result in potent pro- or anti-inflammatory responses. Other endogenous ligands, also present in biological samples, include resolvins, amyloidogenic proteins, such as beta amyloid (Aβ)-42 and prion protein (Prp)_106–126_, the neuroprotective peptide, humanin, antibacterial peptides, annexin 1-derived peptides, chemokine variants, the neuropeptides, vasoactive intestinal peptide (VIP) and pituitary adenylate cyclase activating polypeptide (PACAP)-27, and mitochondrial peptides. Upon activation, intracellular domains of FPR2 mediate signaling to G-proteins, which trigger several agonist-dependent signal transduction pathways, including activation of phospholipase C (PLC), protein kinase C (PKC) isoforms, the phosphoinositide 3-kinase (PI3K)/protein kinase B (Akt) pathway, the mitogen-activated protein kinase (MAPK) pathway, p38MAPK, as well as the phosphorylation of cytosolic tyrosine kinases, tyrosine kinase receptor transactivation, phosphorylation and nuclear translocation of regulatory transcriptional factors, release of calcium and production of oxidants. FPR2 is an attractive therapeutic target, because of its involvement in a range of normal physiological processes and pathological diseases. Here, we review and discuss the most significant findings on the intracellular pathways and on the cross-communication between FPR2 and tyrosine kinase receptors triggered by different FPR2 agonists.

## 1. Introduction

Formyl-peptide receptors 1, 2 and 3 (FPR1, FPR2 and FPR3) form a subgroup of receptors linked to inhibitory G-proteins (G_i_). Their activation by specific agonists leads to transient calcium fluxes, extracellular signal-regulated kinase (ERK) phosphorylation and chemotaxis. FPR2 seems unusual, being used by both lipid and protein ligands. Most of the FPR2 agonists are peptides, with the exception of the eicosanoid, lipoxin A4 (LXA4), and of the synthetic small-molecular weight ligands isolated by compound library screens. A number of these peptides are synthesized based on the sequence of known proteins, but their physiological function and their presence *in vivo* has to be proven. On the other hand, many FPR2 peptide agonists have been identified and purified from living organisms. FPR2 transduces the anti-inflammatory or the neuroprotective effects of LXA4 [1] or humanin [2], but it can also mediate pro-inflammatory responses to serum amyloid A (SAA) and other peptides [3–5]. FPR2 shows the ability to bind microbe-derived peptides, such as those derived from *Helicobacter pylori*[6] and HIV-1 [7], but it can also respond to mitochondrial peptides [8]. The ability of FPR2 to mediate several biological effects may be traced to different receptor domains used by different agonists. Here, we review and discuss the most significant findings on the signaling cascades triggered by contradistinct FPR2 ligands in several cell types, as well as on the cross-communication between FPR2 and tyrosine kinase receptors.

## 2. Microbe-Derived Peptides

Hp(2–20) (AKKVFKRLEKLFSKIQNDK) is a cecropin-like peptide derived from *Helicobacter pylori* (*H. pylori*). It is a chemoattractant in monocytes and stimulates nicotinamide adenine dinucleotide phosphate (NADPH) oxidase-dependent superoxide generation acting via FPR2 and FPR3 [6]. The addition of Hp(2–20) to lymphocytes and monocytes, in a mixture aimed at mimicking the mononuclear cell infiltrate of *H. pylori*—infected gastric tissue—triggers inhibition of natural killer (NK) cell-mediated antitumor cytotoxicity, inhibition of T-cell inducibility by IL-2, downregulation of the CD3ζ and subsequent NK and T-cell death by apoptosis. These inhibitory events are prevented by scavengers of NADPH oxidase-derived oxygen radicals and are, thus, by all probability, explained by the FPR2/FPR3-mediated oxygen radical induction by Hp(2–20) [6]. The cecropin-like peptide also stimulates chemotaxis, migration and proliferation of MKN-28 and AGS gastric epithelial cell lines, which express FPR2 and FPR3 [9]. These effects are mediated by FPR-related downstream signaling pathways, which result in the upregulation of the expression and secretion of vascular endothelial growth factor A (VEGF-A), in p44/p42 MAPK activation and in Akt and signal transducer and activator of transcription 3 (STAT3) phosphorylation. Furthermore, Hp(2–20) accelerates healing of rat gastric mucosa after injury brought about by indomethacin [9], suggesting that it could positively affect the remodeling phase of gastric mucosal healing through FPR2- and FPR3-mediated signaling.

HIV envelope proteins contain domains that interact with FPR2, including at least two sequences in gp120 and two in gp41. A synthetic peptide domain (F peptide), corresponding to amino acid residues 414–434 (EGSDTITLPCRIKQFINMWQE) located in the V4-C4 region of gp120 of the HIV-1 Bru strain, is an inducer of chemotaxis and calcium mobilization in monocytes and neutrophils by using FPR2 as a functional receptor [7]. The activation of monocytes by F peptide also results in downregulation of the cell surface expression of CCR5 and CXCR4 in a protein kinase C (PKC)-dependent manner, suggesting that the activation of FPR2 by a peptide domain derived from HIV-1 gp120 could lead to desensitization of cell response to other chemoattractants [7,10].

Another synthetic peptide (RIHIGPGRAFYTTKN) derived from the linear sequence of the V3 region of the HIV-1 envelope gp120 (MN strain), activates the FPR2 receptor in monocytes and neutrophils [11]. Stimulation of monocytes with V3 peptide results in a reduced response to several chemokines that use multiple cell receptors, presumably through a heterologous desensitization of the receptors. This process may represent an important regulatory element in cell response in the presence of multiple stimulants. V3-dependent heterologous desensitization is associated with the rapid phosphorylation of the chemokine receptor, CCR5, on serine residues via a PKC-mediated signal transduction pathway. Furthermore, V3 peptide mobilizes Ca^2+^ and is a potent chemoattractant for human monocytes and neutrophils, but only weakly for T-lymphocytes [11].

The leucine zipper-like domain, T21/DP107, located in the amino terminus of the ectodomain of gp41, is crucial to the formation of fusogenic configuration of the HIV-1 envelope protein, gp41. The synthetic T21/DP107 peptide (NNLLRAIEAQQHLLQLTVWGIKQLQARILAVERYLKDQ) is a potent stimulant of migration and calcium mobilization in human monocytes, neutrophils and FPR2-transfected HEK293 cells, activating FPR2 with high efficiency [12]. However, it is not clear whether soluble gp41 itself is capable of interacting with FPRs or, alternatively, conjugation with CD4, another fusion receptor of HIV-1, may cause exposure of its domains to interact with these receptors. Although FPRs are not used by HIV-1 for fusion, they may participate in the regulation of host innate immune responses seen in AIDS patients, characterized by an initial stimulation of the immune system in the early stage of the disease and followed by progressive immunosuppression.

N36 peptide (SGIVQQQNNLLRAIEAQQHLLQLTVWGIKQLQARIL), which corresponds to amino acid residues 546–581 in the N-terminal heptad repeat region of HIV-1 gp41, induces directional migration and calcium mobilization in human monocytes and neutrophils by using FPR2 as a functional receptor [13]. The activation of FPR2 by N36 peptide in monocytes also results in heterologous desensitization of chemokine receptors, suggesting that the reduced phagocyte response to chemoattractants seen in AIDS patients may be attributed, at least in part, to this molecular mechanism [13]. In human THP-1 monocytes, primary neutrophils and mouse leukocytes, stimulation of FPR2 with N36 peptide or F peptide induces an increase of endogenous tumor necrosis factor-related apoptosis-inducing ligand (TRAIL) expression, which requires NFκB activation. The increased TRAIL expression in the mice significantly suppress the growth of transplanted mouse liver tumor cells by inducing apoptotic cell death [14]. These data provide novel evidence for the physiologic role of FPR2 and TRAIL in tumor immune surveillance and innate immunity and suggest a novel strategy for cancer therapy.

Mammalian immune cells have the capacity to detect secreted or surface-attached bacterial molecules, (PAMPs; pathogen-associated molecular patterns). Upon activation by PAMPs, the host responds with inflammation and activation of the immune system. FPR2, which responds only weakly to formylated peptides, is activated by *S. aureus* peptide toxins, named phenol-soluble modulins (PSMs), at nanomolar concentrations, stimulating chemotaxis, calcium flux and IL-8 release [15]. Two synthetic PSM peptides utilize FPR2 in neutrophils to produce reactive oxygen species, which in turn trigger inactivation of the peptides [16], suggesting that FPR2 is crucial in staphylococcal infections and may represent an attractive target for new anti-infective or anti-inflammatory strategies (Table 1).

## 3. Endogenous Peptides

### 3.1. Mitochondrial Peptides

The mitochondrial proteins derived by ruptured host cells can be the source of *N*-formylated peptides that can act as chemoattractants for leukocytes. Two peptides (formyl-MLKLIV and formyl-MMYALF) derived from subunit 4 and subunit 6 of human mitochondrial NADH dehydrogenase, respectively, trigger a dramatic increase in the phosphorylation level of ERK1/2, as well as changes in cytosolic calcium concentration through either FPR1 or FPR2 in promyelocytic HL-60 cell lines stably expressing either FPR1 or FPR2 [8]. The mitochondrial human peptide, formyl-MMYALF, is the agonist that more efficiently activates FPR2, eliciting the migration of FPR2-expressing HL-60 cells and the activation of the superoxide generating, NADPH oxidase, which plays a crucial role in the host defense mechanism in phagocytes. A non-formylated peptide fragment (MYFINILTL), derived from mouse NADH dehydrogenase subunit 1, is also an FPR2 agonist [17], and an *N*-formylated esapeptide corresponding to the N-terminus of cytochrome *c* oxidase subunit I (formyl-MFADRW) is an FPR2 and FPR3 ligand [8].

Mitocryptide-2 (MCT-2) is a soluble pentadecapeptide (formyl-MTNIRKSHPLMKIIN), produced from mitochondrial cytochrome *b*, whose N-terminus is formylated [18]. MCT-2 efficiently induces migration and activation of peripheral neutrophils, as well as of neutrophilic/granulocytic cells differentiated from HL-60 cells and promotes phosphorylation of ERK1/2 and an increase in intracellular Ca^2+^ concentration by binding to FPR2 [18]. These signaling events are largely prevented by cells pretreated with pertussis toxin (PTX), which ADP-ribosylates G_i_- and G_o_-type G-proteins and renders them insensitive to receptor regulation. Moreover, in neutrophilic/granulocytic cells, MCT-2 induces β-hexosaminidase release, which is a glycosidase that is released from neutrophilic cells upon stimulation of their activating factors. The enzyme release promoted by MCT-2 is completely inhibited by PTX treatment [18]. The observation that FPR2 specifically and efficiently recognizes cryptide MCT-2 suggests that many peptides produced by the degradation of functional proteins may be associated with unidentified physiological regulations (Table 2).

### 3.2. Amyloidogenic Peptides and Proteins

An important group of FPR2 agonists is formed by at least three amyloidogenic polypeptides, associated with chronic inflammation and amyloidosis. It includes serum amyloid A (SAA), the β-amyloid peptide 42 (Aβ42) and a peptide fragment of the aberrant human prion protein (PrP_106–126_). SAA is a major acute-phase protein produced by hepatocytes, macrophages, endothelial smooth muscle and synovial cells after stimulation with inflammatory cytokines. SAA is normally present in the serum, but its concentration is increased up to 1000-fold in response to a variety of injuries, causing acute-phase responses. In chronic inflammation, SAA can be enzymatically cleaved into fragments that precipitate to form amorphous amyloid fibril deposits with progressive loss of organ function, resulting in amyloidosis [19].

SAA binds efficiently to FPR2 [20], triggering Ca^2+^ mobilization, chemotaxis, production of metalloproteases and cytokines and eliciting the expression of cytokine receptors in monocytes, neutrophils, mast cells and T-lymphocytes [21]. In human monocytes, activation of FPR2 by SAA induces the PTX-sensitive production of cytokine CCL2, the activation of ERK and the induction of cyclooxygenase-2 (COX2), which are required for the production of CCL2 [21]. On the other hand, in human umbilical vein endothelial cells (HUVECs), SAA induces CCL2 production via a PTX-insensitive pathway, as well as ERKs, p38MAPK and c-Jun N-terminal kinase (JNK) activation, which are completely prevented by knock-down of FPR2 [22]. In neutrophils, SAA activates FPR2, inducing the secretion of the pro-inflammatory cytokines, IL-8 [3], while monocytes respond to low concentrations of SAA by producing the pro-inflammatory cytokine, TNF-α, and by releasing the anti-inflammatory cytokine, IL-10, in response to high concentrations of SAA. Signaling cascades induced by SAA include ERKs and p38MAPK activation, which are required for TNF-α and IL-10 production, respectively [23]. In these cells, SAA also stimulates FPR2-mediated matrix-metalloproteinase-9 (MMP-9) upregulation, which requires ERKs phosphorylation, intracellular calcium rise and the activation of NF-κB [24].

**Scheme 1:**
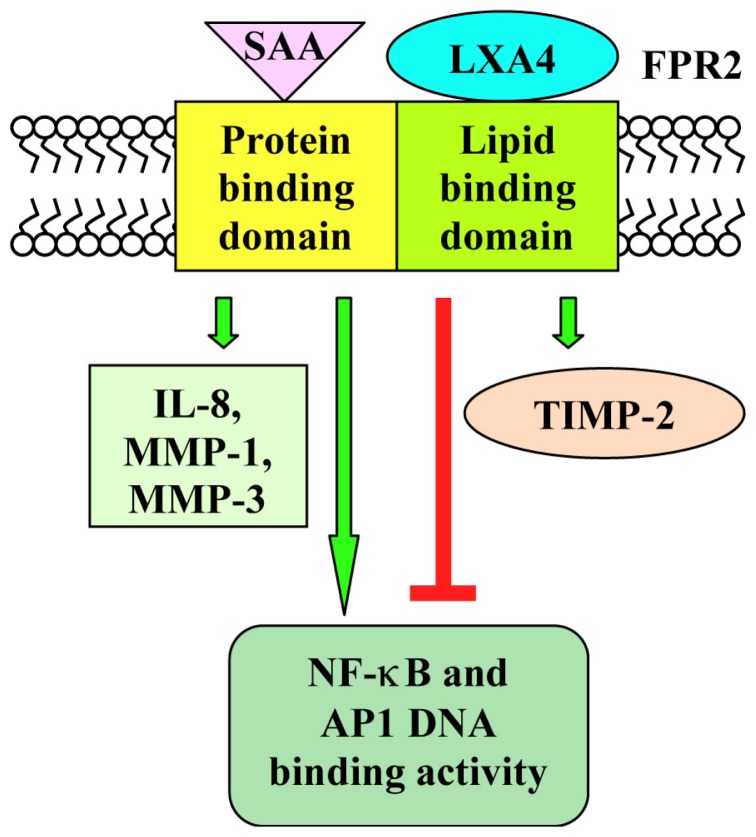
Binding on FPR2 of LXA4 or serum amyloid A (SAA) have opposite effects on metalloproteinase expression and on NF-κB and AP1 DNA binding activity. In human fibroblast-like synoviocytes, SAA promotes the production of metalloproteinase-1 and -3 (MMP-1 and MMP-3) and upregulates NF-κB and AP1 DNA binding activity, by interacting with the protein binding domain of FPR2. On the other hand, LXA4 induces stimulation of tissue inhibitors of metalloproteinase-2 (TIMP-2) and inhibits NF-κB and AP1 DNA binding activity, by interacting with the lipid binding domain of FPR2.

SAA promotes the proliferation of human fibroblast-like synoviocytes (FLS) and protects FLS isolated from patients with rheumatoid arthritis against the apoptotic death induced by serum starvation, anti-Fas IgM and sodium nitroprusside [25]. This activity of SAA depends on the activation of ERKs and Akt and is prevented by downregulation of the FPR2 transcripts with a siRNA [25]. In FLS, SAA also induces the FPR2-dependent expression of metalloproteinase-1 and -3 (MMP-1, MMP1-3) [26] and the production of IL-6 [27]. Although SAA and LXA4 share FPR2 as a functional receptor, they induce different cellular responses in FLS. LXA4 promotes stimulation of tissue inhibitors of metalloproteinase-2, whereas SAA induces IL-8, MMP-1 and MMP-3 production. SAA upregulates NF-κB and Ap1 DNA binding activity, whereas LXA4 markedly inhibits these responses after IL-1β stimulation [28] (Scheme 1). These findings suggest that two endogenous molecules, targeting a common receptor, could participate in the pathogenesis of inflammatory arthritis by differentially regulating inflammatory responses in tissues expressing FPR2 [29].

In human aortic endothelial cells, FPR2 mediates the SAA-dependent production of pentraxin 3 (PTX3), a key component of innate immunity [30]. The action of SAA has been associated also with other receptors, including CD36/LIMPII Analogus-1 [31], toll-like receptor (TLR)-2 [32] and TLR4 [33], and each receptor seems to mediate distinct pharmacological actions. The cytokine-like pro-inflammatory actions occur mainly via FPR2.

The 42-aminoacid form of Aβ42 (DAEFRHDSGYEVHHQKLVFFAEDVGSNKGAIIGLMVGG VVIA) is a self-aggregating peptide produced by sequential cleavage of amyloid precursor protein by the enzymes, β- and γ-secretase. It plays an important role in the pathogenesis of Alzheimer’s disease (AD), exerting its pro-inflammatory responses through FPR2. In this receptor, the N-terminus and a segment between the fourth transmembrane domain and the third intracellular loop are crucial for the interaction with the AD-associated FPR2 agonist [34]. FPR2 gene expression is detected to high levels in CD11b^+^ mononuclear phagocytes surrounding and infiltrating congo-red positive plaques in patients with AD [4]. In microglia and astrocytes cells, Aβ42 is rapidly internalized via FPR2, and phospholipase D (PLD) plays an important role in the regulation of Aβ42-induced endocytosis and FPR2 receptor signaling. FPR2 is recycled to the cell surface with residual antigenic Aβ42 detected in the cytoplasmic region of the cells [35].

Aβ42 induces migration of human monocytes, as well as Ca^2+^ mobilization in FPR2-transfected HEK293 and rat basophile leukemia cells [4]. Furthermore, in a concentration that saturates the chemotactic and calcium flux response, Aβ42 also induces superoxide production in mouse neutrophils and FPR2/HEK293 cell transfectants [36]. Stimulation with Aβ42 of glial cells induces the activation of the PI3K/Akt pathway, which positively regulates ERKs phosphorylation and is dependent on PLD activation [35]. Antagonist- or siRNA-induced receptor inactivation supports the importance of FPR2 for Aβ42-mediated signals transduction in glial cells. In these cells, FPR1, FPR2 and MARCO (macrophage receptor with collagenous structure) show physical and functional interactions necessary to transduce Aβ42 signaling [37].

Brain tissue also produces protective factors that may antagonize the neurodestructive effect of Aβ42. Humanin (HN) is a 24-aa residue neuroprotective polypeptide (MAPRGFSCLLLLTSEID LPVKRRA) expressed in the occipital region of the brain in AD that protects neuronal cells from damage by Aβ42 [38–40]. HN transcripts are also present in heart, skeletal muscle, kidney, liver, brain and gastrointestinal tract. HN induces Ca^2+^ mobilization and chemotaxis in mononuclear phagocytes by using FPR2, the same functional receptor used by Aβ42 to chemoattract and activate phagocytic cells [38]. Furthermore, HN reduces the aggregation and fibrillary formation by suppressing the effect of Aβ42 on mononuclear phagocytes and protects neuronal cells from cytotoxicity and apoptosis caused by Aβ42, suggesting that HN may exert its neuroprotective effects by competitively inhibiting the access of FPR2 to Aβ42 [38]. The intracellular signaling cascade triggered by HN through FPR2 activation also includes ERKs phosphorylation, which is significantly inhibited in cells treated with PTX. However, in F11 neurohybrid cells, siRNA-mediated disruption of expression of mouse FPR2 (mFPR2) does not result in attenuation of HN-mediated rescue of neuronal cell death induced by various AD-related insults. In these cells, neuroprotection by HN is mediated by the STAT3 transcription factor, as well as by certain tyrosine kinases [41], suggesting the involvement of a receptor other than mFPR2. Taken together, these results indicate that mFPR2 is not required for HN-mediated neuroprotection against AD-related insults, even though HN is an efficient agonist for FPR2.

FPR2 is involved in pro-inflammatory processes of prion disorders, which, similar to AD, include the infiltration and activation of mononuclear phagocytes in brain lesions [44]. The etiological agent of Creutzfeldt-Jakob disease in humans is an aberrant isoform of the cell surface glycoprotein, the prion protein (PrPc). The pathologic isoform of PrPc (PrPSc) is deposited in the extracellular space of diseased CNS at sites infiltrated by activated astrocytes and microglia. PrP_106–126_ is a 21-amino acid fragment (KTNMKHMAGAAAAGAVVGGLG) of the aberrant human protein, which can form fibrils *in vitro* and can induce several biological responses. In glial cells, PrP_106–126_ binds efficiently to FPR2, triggering protein tyrosine phosphorylation, an increase of pro-inflammatory cytokines (IL-6, TNF-α), implicated as neurotoxic mediators, PTX-sensitive calcium mobilization and chemotaxis [42]. PrP1_06–126_ shows chemotactic activity also on the macrophage cell line, Ana-1, which is prevented by PTX and Tyrphostin-23, suggesting the involvement of G_o_/G_i_ proteins and members of the Src-family tyrosine kinase [43]. Similar to Aβ42, in astrocytes and microglia, the internalization of PrP_106–126_ is mediated by FPR2 [45] (Table 3).

### 3.3. Peptides Associated with Inflammatory and Anti-inflammatory Responses

Urokinase-type plasminogen activator (uPA) is a serine protease that activates plasminogen into plasmin. It binds to a specific high affinity cell surface receptor (uPAR), thereby inducing intracellular signaling that affects cell adhesion, cell migration and proliferation. uPAR contains three extracellular domains connected by a linker of 15–20 amino acids each. The N-terminal D1 domain interacts with uPA, D2 connects D1 and the *C*-terminal D3 domain contains a glycosylphosphatidylinositol anchor. uPA cleaves the D1-D2 linker region, generating a soluble D2D3_88–274_ fragment that binds and activates FPR2, inducing cell migration [5]. The cleaved soluble uPAR activates other members of the FPR family. For instance, SRSRY, a peptide corresponding to residues 88 to 92 of uPAR, binds and activates FPR1 [46], and uPAR_84–95_ induces basophil migration by activating both FPR2 and FPR3 [47].

Pretreatment of monocytes with the FPR2 agonist, D2D3_88–274_, markedly decreases chemokine-induced integrin-dependent rapid cell adhesion [48], indicating that FPR2 regulates leukocyte chemotaxis as a direct mediator of cell migration and/or by suppressing cell responses to chemokines by desensitizing chemokine receptors [48]. uPAR works in concert with co-receptors, including integrins, FPR2 and epidermal growth factor receptor (EGFR), which may be dynamically organized into a multiprotein signaling receptor complex to initiate cell signaling [49]. At least two forms of uPAR are present on the cell surface, full-length and cleaved uPAR, each specifically interacting with one or more transmembrane proteins. The uPAR mutant, hcr (human cleavage resistant), is not cleaved by proteases, is glycosylphosphatidylinositol anchored and binds uPA. Both wild-type (wt) and hcr-uPAR are able to mediate uPA-induced migration, are constitutively associated with the EGFR and associate with integrins upon uPA binding. However, they engage different pathways in response to uPA. wt-uPAR requires both integrins and FPR2 to mediate uPA-induced migration, and association of wt-uPAR to integrin results in uPAR cleavage and ERK activation. On the contrary, hcr-uPAR does not activate ERK and does not engage FPR2, but it activates an alternative pathway initiated by the formation of a triple complex (uPAR-integrin-EGFR) and resulting in the auto-tyrosine phosphorylation of EGFR [50].

Formyl peptide receptors interact with bactericidal peptides. hCAP18 belongs to a family of proteins called cathelicidins, which usually consist of a highly conserved pre-proregion of 128–143 residues, including a putative 29–30-residue signal peptide, a 99–114-residue cathelin-like domain and a COOH-terminal antimicrobial domain ranging in length from 12 to >100 amino acid residues. Cleavage of hCAP18 occurs between Ala103 and Leu104, giving rise to LL-37 (LLGDFFRKSKEKIGKEFKRIVQRIKDFLRNLVPRTES), a 37-residue mature antimicrobial peptide with two leucine residues on its NH_2_ terminus. LL-37/hCAP18 is produced by neutrophil granules and various epithelial cells and secreted into wound and airway surface fluid.

LL-37 is chemotactic and induces Ca^2+^ mobilization in human monocytes and FPR2-transfected HEK293 cells, and these responses can be cross-desensitized by an FPR2-specific agonist. LL-37 is also chemotactic for human neutrophils and T-lymphocytes that are known to express FPR2, suggesting that LL-37 may contribute to innate and adaptive immunity by recruiting neutrophils, monocytes and T-cells to sites of microbial invasion by interacting with FPR2 [51]. LL-37 suppresses neutrophil apoptosis, which is attenuated by the antagonists for FPR2 and P2X7 nucleotide receptor and requires ERKs phosphorylation, expression of the anti-apoptotic protein, Bcl-xL, and inhibition of caspase 3 activity [52]. On the other hand, in these cells, LL-37 specifically inhibits SAA-induced IL-8 production and chemotactic migration and causes a dramatic inhibition of ERK and p38MAPK activity, which is induced by this acute-phase protein. The LL-37-induced inhibitory effect is mediated by FPR2 [53]. Application of LL-37 in different *in vivo* models for angiogenesis and arteriogenesis results in a significant induction of vessel growth, which is mediated by FPR2 expressed on endothelial cells. The involvement of FPR2 in endothelial stimulation by LL-37 is supported by inhibition studies using PTX and a neutralizing antiserum to FPR2, which completely block the proliferative effect of LL-37. Downstream events of FPR2 activation include the PTX-sensitive increase of intracellular Ca^2+^ and nuclear translocation of NF-κB. This latter is prevented by the PKC-inhibitor, GF109203X, which also abolishes the LL-37–induced increase in proliferation, and by the antioxidant, N-acetylcysteine, indicating the involvement of reactive oxygen species. The mitogen-activated protein kinase kinase (MEK) inhibitor, PD098059, also prevents LL-37-mediated endothelial proliferation, suggesting that ERKs may be involved in LL-37-elicited effects [54].

LL-37 also stimulates healing of mechanically induced wounds, as well as cell proliferation and migration, in the bronchial mucoepidermoid carcinoma-derived cell line, NCI-H292, and in differentiated primary airway epithelium. Inhibitory studies indicate that these effects are likely mediated by FPR2, since PTX inhibits wound healing significantly [55]. Several pieces of evidence support an anti- and pro-tumorigenic role for LL-37. In mesenchymal stromal cells (MSCs), LL-37 significantly reduces the engraftment of these cells into ovarian tumor xenografts, resulting in inhibition of tumor growth, as well as disruption of the fibrovascular network. Migration and invasion experiments indicate that the LL-37-mediated migration of MSCs to tumors occurs through FPR2, being prevented by PTX. In these cells, LL-37 also induces PTX-sensitive ERKs phosphorylation, providing further evidence in support of notion that LL-37 stimulates MSCs through FPR2 [56]. LL-37 induces invasion in ovarian cancer cells and stimulates MAPK and JAK/STAT signaling cascades, as well as the significant activation of several transcription factors, through both FPR2-dependent and FPR2-independent pathways. A gene microarray has shown the expression profiles of genes regulated by the LL-37–FPR2 interaction in ovarian cancer. These include angiopoietin-like 3, complement 5 (C5), collagen type XVIII, epidermal growth factor (EGF), fibroblast growth factor 1 (FGF1), FPR2, hCAP-18/LL-37, the matrix metalloproteinases 2 (MMP-2) and uPA. LL-37-stimulated genes are attenuated by the inhibition of FPR2, suggesting that LL-37 potentiates a more aggressive behavior from ovarian cancer cells through its interaction with this receptor [57]. Cancer cells recruit monocytes, macrophages and other inflammatory cells by producing abundant chemoattractants and growth factors, such as macrophage colony-stimulating factor (M-CSF/CSF-1) and monocyte chemoattractant protein-1 (MCP-1/CCL2), to promote tumor growth and dissemination.

LL-37 stimulates M-CSF and MCP-1 expression in human and mouse hepatocellular carcinoma cells, and these effects are dependent on the activation of FPR2 and subsequent ROS-MAPK-NF-κB signaling [58]. LL-37 and leukotriene B4 (LTB4) are important pro-inflammatory mediators. LTB4 triggers LL-37 release from human neutrophils (PMNs) and, conversely, LL-37 promotes LTB4 production from these cells. The effect of LL-37 is mediated by FPR2, and the signal transduction leading to LTB4 release involves p38MAPK and phosphorylation of cPLA2 [59,60].

**Scheme 2:**
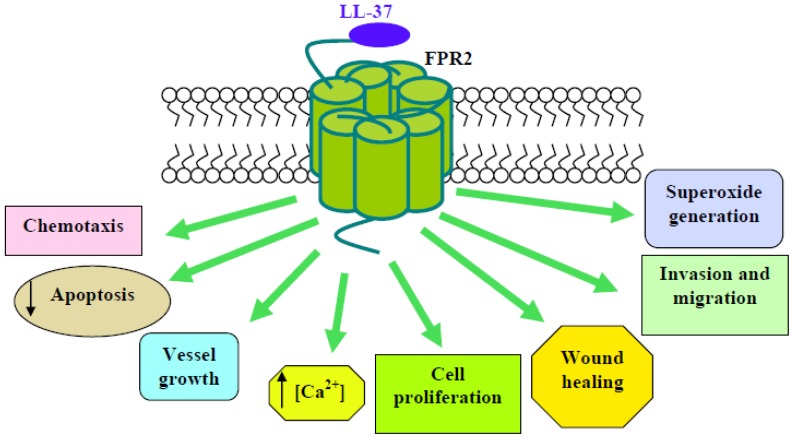
Intracellular events triggered after FPR2 activation by LL-37. In human monocytes, neutrophils and T-lymphocytes, LL-37 induces chemotaxis and Ca^2+^ mobilization and suppresses apoptosis. In endothelial cells and in different *in vivo* models for angiogenesis and arteriogenesis, application of LL-37 results in a significant induction of vessel growth and in an increase of cell proliferation. LL-37 also stimulates wound healing, invasion and migration in the bronchial mucoepidermoid carcinoma-derived cell line, NCI-H292, and in differentiated primary airway epithelium. In human fibroblasts, IMR90, LL-37 induces NADPH oxidase-dependent superoxide generation and p47^phox^ phosphorylation.

In human fibroblasts, IMR90 LL-37 induces NADPH oxidase-dependent superoxide generation, p47^phox^ phosphorylation and translocation and ERKs phosphorylation, which are prevented by PTX, by the FPR2 antagonist, WRWWWW (WRW4), by the MEK inhibitor, PD098059, and by calcium depletion [61] (Scheme 2).

FPR2 interacts with a chemokine variant, activating phagocytic leukocytes. Myeloid progenitor inhibitor factor-1 (CCL23/MPIF-1) belongs to the CC(β) subfamily of cytokines, and its cDNA encodes a 99 (CKβ8) or 116 amino acid (CKβ8-1) mature form protein. Both splice variants have been identified as putative ligands for CCR1 receptor. An N-terminal truncated form (amino acids 22–137) of CKβ8-1 (sCKβ8-1) elicits a dose-dependent increase in the mobilization of intracellular calcium in FPR2-expressing CHO-K1 cells, as well as chemotaxis in polymorphonuclear (PMN) leukocytes [62]. The generation of sCKβ8-1 involves proteases associated with inflammation that cleave CCL23 immediately from the N-terminal to the 18-residue domain encoded by the alternatively spliced nucleotides [63]. The proteases also cleave CCL23 immediately from the C-terminal to the inserted domain, producing a typical CC chemokine “body” containing even further-increased CCR1 potency and a 18-amino acid peptide (MLWRRKIGPQMTLSHAAG), termed SHAAGtide, with full FPR2 activity, but no activity for CCR1. In human monocytes and neutrophils, SHAAGtide induces calcium mobilization and chemotaxis, mediated by FPR2 [63]. This suggests an intriguing molecular mechanism by which protease cleavage of a chemokine produces two peptides acting on two different receptors. However, the presence of the cleaved products *in vivo* has not yet been proven.

The vasoactive intestinal polypeptide (VIP) is a pleiotropic peptide produced by neurons in different areas of the CNS and by endocrine cells. VIP interacts with the VIP/pituitary adenylate cyclase-activating protein (VPAC1) receptor, a G-protein coupled receptor (GPCR) constitutively expressed in human resting T-cells, neutrophils, monocytes, macrophages, dendritic cells and bone marrow stromal cells [64]. VIP/VPAC1 binding activates the cAMP/protein kinase A (PKA) pathway, which is the major mediator of VIP effects on hematopoıetic cells, although VIP-induced cAMP-independent pathways have also been reported. In cells of hematopoietic and non-hematopoietic origin, VIP has anti-inflammatory [65] and pro-inflammatory properties [66]. The pro-inflammatory effect of VIP is mediated via the VPAC1 receptor and FPR2. VIP/VPAC1 interaction is associated with a cAMP increase and activation of the cAMP/p38MAPK and cAMP/EPAC/PI-3K/ERK pathways, which regulate MMP-9, CD35 and CD11b exocytosis or CD11b expression, respectively; VIP/FPR2 interaction results in cAMP-independent PI3K/ERK activation with downstream integrin upregulation [67]. The role of G_αi_ in this mechanism is supported by the observation that PTX prevents Akt and ERK phosphorylation and inhibits CD11b upregulation [67]. These observations suggest that the pro-inflammatory effects of VIP lie behind different receptor interactions and multiple signaling pathways.

The two pituitary adenylate cyclase-activating polypeptides (PACAPs), PACAP27 (HSDGIFTDSYSRFRKQMAVKKLAAVL) and PACAP38, are neuropeptides that belong to the VIP family [68]. PACAPs are multifunctional peptide hormones that influence diverse biological functions and that suppress and activate inflammation by regulating IL-1β, IL-6 and IL-10. In human neutrophils, PACAP27 stimulates intracellular calcium mobilization, CD11b surface upregulation, chemotactic migration and ERK, Akt and p38MAPK phosphorylation by activating FPR2. The molecular responses elicited by the neuropeptide are prevented by WRW4, thus supporting the notion that PACAP27 acts on FPR2 [69]. PACAP27-evoked Ca^2+^ mobilization involves both Ca^2+^ influx and intracytoplasmic Ca^2+^ release through PTX-, PKA- and adenylate cyclase-dependent mechanisms [70] (Table 4).

### 3.4. Annexin A1 and Derived Peptides

Annexin A1 (ANXA1) is a glucocorticoid-regulated phospholipid-binding protein of 37 KDa with pro- and anti-inflammatory activity, mediated in part by FPRs activation [71]. ANXA1 is particularly abundant in neutrophils, but it is also expressed in a variety of cell types. The protein is externalized onto the neutrophil cell surface, where it acts by inhibiting transendothelial migration. The pro- and anti-inflammatory activities elicited by ANXA1 are mediated by peptides derived from its N-terminus domain, which are most likely generated at sites of inflammation. The bioactive N-terminus domains (Ac2-26; Ac-AMVSEFLKQAWFIENEEQEYVQTVK and Ac9-25; Ac-QAWFIENEEQEYVQTVK) plays significant roles in the inhibition of adhesion and transmigration of leukocytes, thereby limiting the intensity and duration of the inflammatory response and promoting proliferation and invasion in epithelial cells [72]. ANXA1 and Ac2-26 bind formyl-peptide receptors [71,73] with a different affinity constant, influenced by the selective affinity of ANXA1 to FPR2 compared to Ac2-26, which appears to be a more promiscuous ligand for members of the FPRs family [74]. At high concentrations, the ANXA1 peptides show a potent pro-inflammatory activity and activate neutrophils *in vitro* by acting on FPR1. In contrast, other studies show that these peptides use FPR2 for their anti-inflammatory actions [75], as in the case of the ANXA1 core-derived peptide antiflammin-2 (HDMNKVDK), which binds efficiently to FPR2 in FPR2-transfected HEK293 cells, triggering ERKs phosphorylation [76]. The core structure that binds FPR1 and activates NADPH oxidase to release superoxide anion is the sequence Gln9-Ala10-Trp11-Phe12 in the N-terminus region of ANXA1 [77]. Early intracellular phosphorylation events seem common to receptor activation by ANXA1 and bioactive peptides. In fact, in polymorphonuclear leukocytes (PMN) and in HEK-293 cells transfected with FPR1 or FPR2, stimulation with Ac2-26 triggers ERKs phosphorylation via both FPR1 and FPR2, and ANXA1 binds FPR2 only in cells transfected with FPR2, eliciting ERKs activation [73].

In synovial fibroblasts, ANXA1 and Ac2-26 induce TNFα-stimulated matrix metalloproteinases-1 (MMP-1) secretion, which is inhibited by PTX and by a specific FPR2 antagonist [78]. Several studies have described the effects of ANXA1 and Ac2-26 on cell proliferation and invasion. In breast epithelial tumor cells, the signaling elicited by ANXA1/FPR2 interaction induces an increased level of cyclin D1, which is associated to the activation of the PI3K/Akt/p70S6K pathway [79], and in SKCO-15 colorectal adenocarcinoma, ANXA1 mediates cell invasion via FPR2 [80]. Furthermore, Ac2-26 stimulates proliferation of MDA-MB-231 breast tumor cell lines [81], and ANXA1 regulates TNFα-induced proliferation and inflammatory responses in lung fibroblasts, via effects on the ERK and NF-κB pathways [82]. These responses depend on FPR2 activation, being prevented by incubation with the FPR2 antagonist, WRW4, or by a siRNA against FPR2 and suggest a role for FPR2 signaling in cancer cell mitogenesis and invasion. The FPR2 N-terminus domain conveys ANXA1 signaling through calcium mobilization and ERK phosphorylation, whereas the second extracellular loop is required to provoke more sustained changes, such as those leading to modulation of gene expression. This suggests that at least two FPR2 sites are required to accommodate this agonist [83], even though a three-point binding model [84], where binding sites are within the non-conserved amino acid residues, 84, 85 (point 1), 163 (point 2) and 284 (point 3), has also proposed (Table 5).

### 3.5. Other Endogenous Peptides

Temporin A (TA; FLPLIGRVLSGIL), a frog-derived antimicrobial peptide, binds efficiently to FPR2, stimulating PTX-sensitive migration of human monocytes, neutrophils and macrophages, as well as p44/42 MAPK activation and Ca^2+^ flux in monocytes [85]. TA is also chemotactic *in vivo*, because it elicits infiltration of neutrophils and monocytes into the injection site in mice. Another three temporin peptides (I4S10-C; FLPIIASLLSKLL, I4G10-C; FLPIIASLLGKLL and Rana-6; FISAIASMLGKFL) induce the migration of FPR2-transfected HEK293 cells, suggesting that these antimicrobial peptides also use FPR2 as a chemotactic receptor [85]. The biological significance of these observations is unclear, because temporin homologs have not been isolated in mammals and FPRs have not been characterized in frogs.

ApoE and apoA-I, major protein components of circulating high-density lipoprotein (HDL) particles, have anti-inflammatory effects on several cell types in the cardiovascular system. Synthetic peptides have been designed that mimic the class A amphipathic α-helical domains of apoA-I and apoE and that retain the anti-inflammatory activity [86]. In human PMNs, monocytes and in FPR2-transfected HEK293 cells, the apoA-I mimetic peptide L-37pA (DWLKAFYDKVAEKLKEA FPDWLKAFYDKVAEKLKEAF) induces calcium flux and chemotaxis through FPR2, whereas its d-stereoisomer blocks L-37pA signaling. L-37pA could represent a novel chemotactic agent, which possesses a complex structure-activity relationship and which displays anti-inflammatory efficacy against innate immune responses in the airway [86] (Table 6).

## 4. Endogenous Nonpeptide Ligands

FPR2 shows the unusual feature of recognizing both lipid and protein agonists. Lipoxins are lipid mediators generated at sites of inflammation by the sequential action of 5 and 12 or 15 and 5 lipoxygenase, depending on the cellular context. Lipoxin A4 (LXA4; 5*S*,6*R*,15*S*-trihydroxy-7,9,13- *trans*-11-eicosatetraenoic acid) is an unusual metabolite of arachidonic acid with anti-inflammatory and immunoregulatory biological functions [87]. It exerts its effects by binding FPR2 with high affinity, thereby stimulating arachidonate release and GTPase, cPLA2 and PLD activities [88–92]. LXA4 blocks neutrophil infiltration and transmigration across mucosal epithelial cells and vascular endothelial cells [93,94] through induction of NO production, which suppresses leukocyte-endothelial cell interaction [95]. LXA4 also interacts directly with the CysLT1 receptor and induces signals that prevent the pro-inflammatory responses, which contribute to regulating the resolution of inflammation [87]. The seventh transmembrane domain and adjacent regions of the FPR2 receptor are essential for LXA4 recognition, whereas additional regions of FPR2 (e.g., extracellular loops) are required for high-affinity binding of peptide ligands. These findings support the findings that FPR2 can recognize specific chemotactic peptides, as well as lipid-derived ligands, but with different affinity and/or distinct interaction sites within the receptor [17]. Probably, one of the mechanism of switching receptor functions is deglycosylation of FPR2, which does not dramatically alter LXA4 recognition, but significantly lowers the affinity for peptide ligands [17].

In human neutrophils, FPR2 activation by LXA4 stimulates intracellular Ca^2+^ increase, chemotaxis and PKC-dependent PLD activation, but doesn’t trigger the activation of MAPK and cPLA2, which is required for NADPH oxidase-dependent superoxide generation and is usually elicited in neutrophils by FPR peptide ligands [96]. In human renal mesangial cells (MC), which express receptors for both LXA4 and leukotriene D4 (LTD4), LXA4 inhibits LTD4-induced PI3K activity and MC proliferation [97]. LTD4 stimulates ERKs and p38MAPK via a PTX-sensitive pathway that depends on PI3K and PKC activation. Unlike LTD4, LXA4 activation of ERKs is insensitive to PTX and PI3K inhibition, whereas LXA4 activation of p38MAPK is sensitive to PTX and can be blocked by a LTD4 receptor antagonist [97]. These data suggest that LXA4 stimulation of the MAPK superfamily involves two distinct receptors: one shared with LTD4 and coupled to a PTX-sensitive G-protein and another coupled via an alternative G-protein, such as G_q_ or G_12_, to ERK activation. The cascade by which LXA4 prevents LTD4-induced PI3K activation is mediated by modulation of receptor tyrosine kinase activity through LXA4-induced inhibition of platelet-derived growth factor (PDGF)-Rβ and EGF-R [98]. LXA4 also modulates PDGF-induced decrements in the levels of the cyclin/cdk complex inhibitors, p21^cip1^ and p27^kip1^, and inhibits the PDGF-induced increases of CDK2-cyclin E complex [17]. Inhibition of PI3K mimics the effects of LXA4 with respect to nuclear retention of p27^kip1^, suggesting that LXA4 modulates PDGF-induced proliferation by attenuating Akt activation and preventing G1-S progression [98]. These antiproliferative effects are mediated by FPR2, since serum-stimulated proliferation of FPR2-expressing CHOK1 cells is attenuated by LXA4 analogs [98]. The role of LXA4 in cell proliferation has been also observed in human lung fibroblasts (HLF), stimulated by connective tissue growth factor (CTGF) [99]. In these cells, which express FPR2, CTGF induces cell proliferation, ERKs, PI3K and PKB phosphorylation, enhances the expression of cyclin D1, stimulates STAT3 DNA-binding activity and inhibits the expression of p27^kip1^[99]. LXA4 downregulates the responses elicited by CTGF. Pretreatment with PTX blocks the inhibitory effects of LXA4 on CTGF-induced proliferation, and overexpression of FPR2 enhances the inhibitory effects of LXA4, suggesting that FPR2 mediates the responses exerted by LXA4 on HLF [99]. In MCF-7 and MDA-MB-231 breast tumor cell lines, stimulation of FPR2 by LXA4 induces an increase in cyclin D1 protein and in phosphorylation of Akt and p70S6K. These responses are prevented by the PI3K inhibitor LY294002 and attenuated by FPR2 antagonism [81].

Stimulation of human synovial fibroblasts with IL-1β upregulates the expression of FPR2 and induces the synthesis of IL-6, IL-8 and matrix metalloproteinase-1 and -3. LXA4 inhibits these IL-1β responses and prevents metalloproteinase-3 synthesis without significantly affecting metalloproteinase-1 levels. Inhibition by LXA4 of IL-1β-induced IL-8 synthesis is abrogated by an anti-FPR2 antibody, suggesting that LXA4 may regulate classical IL-8 gene transcription pathways via FPR2 [100]. Part of the anti-inflammatory effect of LXA4 involves the inhibition of NF-κB and AP1, which are responsible for the expression of many pro-inflammatory cytokines and chemokines. In fact, LXA4 downregulates IL-1β-induced AP1 and NF-κB DNA binding complexes at subnanomolar concentrations, which is in accordance with its affinity for FPR2 [101]. Whereas LXA4 suppresses the expression of these pro-inflammatory cytokines, it stimulates the FPR2-dependent expression of the anti-inflammatory molecule, IL-10 [102], and of the antioxidant molecule, heme oxygenase1 [103]. LXA4 treatment inhibits FPR2-dependent pro-inflammatory cytokine production also in bronchial [104] and intestinal epithelial cells [105]. In dendritic cells, LXA4 binds on FPR2, exerting its anti-inflammatory effect through induction of the suppressor of cytokine signalling-2 [106].

Resolvins, protectins and maresins are novel lipid mediators in the resolution of inflammation. They are synthesized as transcellular events from arachidonic acid and omega-3 fatty acids, such as docosahexaenoic acid (DHA) and eicosapentaenoic acid (EPA). Enzymes involved in the biosynthesis of these mediators include COX2, aspirin-induced acetylated COX2, 5-lipoxygenase (5-LO), 12-LO and 15-LO [107].

These pro-resolving mediators include the E-series resolvins (RvE1 and RvE2), which are derived from EPA, the D-series resolvins (RvD1–D6), the neuroprotectins/protectins and maresin, which is derived from DHA [111]. They promote several actions mediated by various membrane receptors. RvD1 (7*S*,8*R*,17*S*-trihydroxy-4*Z*,9*E*,11*E*,13*Z*,15*E*,19*Z*-docosahexaenoic acid) interacts with the phagocyte GPCR-32 receptor as a potent agonist to signal for pro-resolving responses and can directly activate FPR2 with high affinity. The other D-series resolvins demonstrate structure-activity relationships indicative of receptor-mediated signaling pathways, but these receptors have not yet been identified [112]. The RvD1 receptor, FPR2, is expressed in fresh, isolated cells from mouse salivary glands and in cell lines of salivary origin. RvD1 stimulation abolishes tight junction and cytoskeletal disruption caused by TNF-α and enhances cell migration and polarity in salivary epithelium. These effects are blocked by the FPR2 antagonist, butyloxycarbonyl-Phe-Leu-Phe-Leu-Phe, and are mediated by the FPR2-dependent modulation of the PI3K/Akt pathway, as observed by the effects of LY294002 and Akt gene silencing. This suggests that FPR2 activation by RvD1 promotes resolution of inflammation and tissue repair in salivary epithelium, which may have relevance in the restoration of salivary gland dysfunction associated with Sjögren’s syndrome epithelium [108]. In acute lung injury, FPR2 activation by RvD1 significantly decreases levels of pro-inflammatory cytokines, including IL-1β, IL-6 and TNF-α, and decreases NF-κB-phosphorylated p65 nuclear translocation [109]. The same molecular responses are observed in inflamed obese adipose tissue, where RvD1 rescues impaired expression and secretion of adiponectin and decreases pro-inflammatory adipokine production, including leptin, TNF-α, IL-6 and IL-1β [110] (Table 7).

## 5. Ligands from Peptide Library

The first molecule proposed as a specific FPR2 agonist is the WKYMVm (Trp-Lys-Tyr-Met-Val-d-Met) peptide. It was isolated by screening a synthetic peptide library composed of random sequences of hexapeptides and contains a d-methionine at position 6 that greatly enhances its biological activity [113]. WKYMVm binds to FPR2 with high efficiency and with lesser efficiency to FPR1 and FPR3 [114–118], thereby activating neutrophil and monocyte functions, including chemotaxis, mobilization of complement receptor-3, cytokine release and activation of NADPH oxidase, which results in the respiratory burst [116–118]. WKYMVm activates neutrophils through FPR1 only when signaling through FPR2 is blocked, which is indicative of a receptor switch [119].

In human neutrophils, WKYMVm promotes an increase of intracellular calcium concentration, NADPH oxidase activation, cPLA2-mediated arachidonic acid release and an increase of LTB4 production, acting selectively on FPR2 [115,120]. In monocytes, binding of WKYMVm to FPR2 activates chemotaxis associated to the phosphorylation of several cellular proteins, including p125FAK, Pyk, MEK, ERKs, Akt and RhoA [118], and stimulates NADPH oxidase-dependent superoxide generation through PKC and PLD activation [121]. In eosinophils, the hexapeptide induces ERKs phosphorylation and superoxide production via a PI3K-mediated ERKs pathway [122]. Stimulation of U937 cells with WKYMVm activates ERKs via a G-protein/PI3K/Ras/Raf-1 mediated signaling pathway [123] and enhances cPLA2 and PLD activation, triggering lysophosphatidic acid (LPA) formation. The inhibition of LPA synthesis by n-butanol or a cPLA2-specific inhibitor significantly prevents WKYMVm-induced Ca^2+^ influx, suggesting a crucial role for LPA in this process [124]. The stimulation with WKYMVm elicits chemotactic migration of IL-2-activated NK cells, but not resting NK cells, which is completely inhibited by PTX. WKYMVm also stimulates ERK, p38MAPK and JNK activities in both resting and IL-2-activated NK cells. Chemotactic migration is partially prevented by the MEK inhibitor, PD098059, and by the selective FPR2 antagonist, WRW4 [125].

**Scheme 3:**
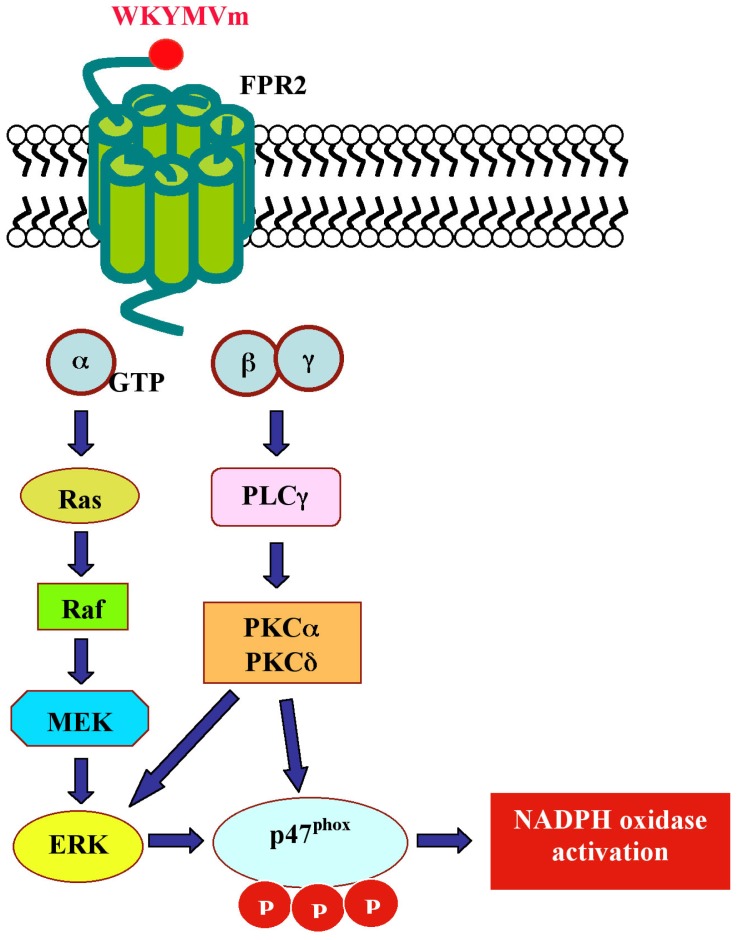
Intracellular signaling pathways elicited by WKYMVm in human fibroblasts, IMR90. The exposure of human fibroblasts, IMR90, to WKYMVm induces ERKs activation, p47^phox^ phosphorylation and NADPH oxidase activation. Superoxide generation by WKYMVm in these cells requires also the activation of PKCα and PKCδ, which translocate from the cytosolic to the membrane fraction upon stimulation with the hexapeptide.

**Scheme 4:**
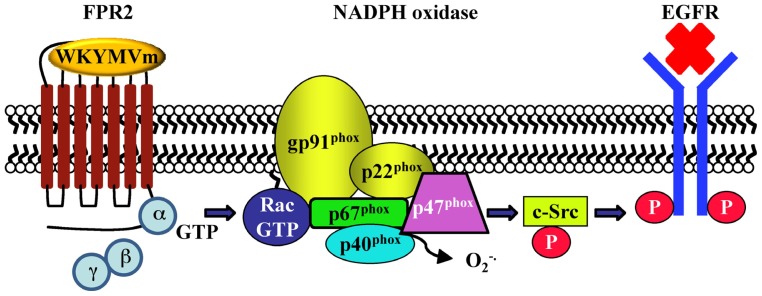
The cross-talk between FPR2 and EGFR is mediated by NADPH oxidase-dependent superoxide generation and by c-Src activation. Stimulation of CaLu-6 cells with WKYMVm induces p47^phox^ phosphorylation and translocation, NADPH oxidase activation, c-Src kinase activity and EGFR transactivation. Oxidation of the cysteine sulfhydryl group of phosphotyrosine phosphatase (PTPase) by reactive oxygen species tightly controls the activity of EGFR, shifting the equilibrium state of EGFR from non-phosphorylated to phosphorylated. c-Src is also sensitive to intracellular redox conditions and plays a key role in bridging signals from FPR2 to EGFR in these cells. NADPH oxidase-dependent superoxide generation can inactivate PTPases that control the c-Src phosphorylation status.

**Scheme 5:**
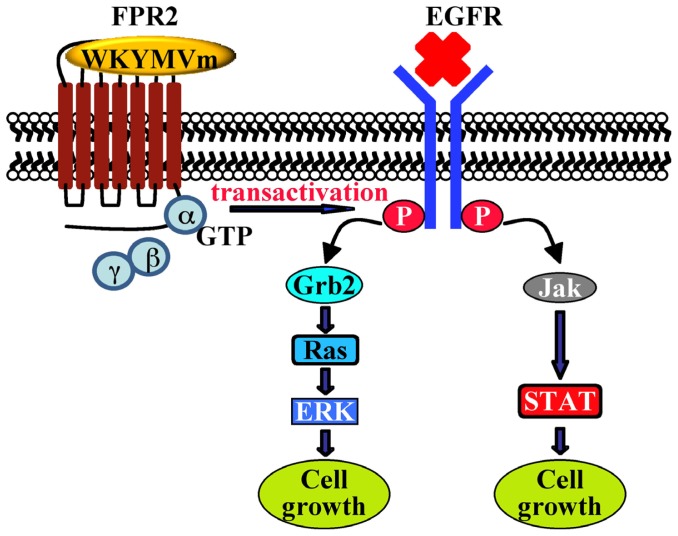
Cross-talk between FPR2 and EGFR plays an instrumental role in orchestrating downstream signaling molecules. In CaLu-6 cells exposed to WKYMVm, the FPR2-dependent EGFR transactivation results in the phosphorylation of critical tyrosine residues, which provide docking sites for recruitment and triggering of Ras/ERK and Jak/STAT pathways. The cellular response to FPR2-induced signaling is an increase of cell growth.

Human fibroblasts, IMR90, express FPR2. The exposure to WKYMVm induces ERKs activation, p47^phox^ translocation and NADPH-dependent superoxide generation, and these effects are in large part prevented by preincubation with the MEK inhibitor and by PTX. HEK293 cells, which express a NADPH oxidase-like enzyme, but not formyl peptide receptors, transiently transfected with FPR2 cDNA generate superoxide on stimulation with WKYMVm, demonstrating that FPR2 is a biologically functional receptor in these cells [126]. NADPH oxidase-dependent superoxide generation by WKYMVm in IMR90 cells requires also the activation of PKCα and PKCδ which translocate from the cytosolic to the membrane fraction upon stimulation with the hexapeptide [127] (Scheme 3). In human lung cancer CaLu-6 cells, stimulation with WKYMVm induces EGFR tyrosine phosphorylation, p47^phox^ phosphorylation, NADPH-oxidase-dependent superoxide generation and c-Src kinase activity (Scheme 4). As a result of EGFR transactivation, phosphotyrosine residues provide docking sites for recruitment and triggering of the STAT3 pathway. WKYMVm-induced EGFR transactivation is prevented by WRW4, PTX and a c-Src inhibitor. The critical role of NADPH-oxidase-dependent superoxide generation in this cross-talk mechanism is corroborated by the finding that apocynin or a siRNA against p22^phox^ prevents EGFR transactivation and c-Src kinase activity. In addition, WKYMVm promotes CaLu-6 cell growth, which is prevented by PTX and WRW4 [128] (Scheme 5).

The activation of FPR2 by WKYMVM in human U87 astrocytoma and FPR2-transfected CHO cells triggers JNK, ERKs and p38MAPK phosphorylation. The key signaling intermediates in the MAPK pathways include G_i_/G_o_ proteins and Src family tyrosine kinases, which are required to transmit signals from FPR2 toward JNK, ERKs and p38MAPK and PLCβ, which is involved in the WKYMVm-induced regulation of JNK. The FPR2-activated MAPKs mediate glial fibrillary acidic protein (GFAP) and IL-1α upregulation, which is correlated with reactive astrocytosis [129]. The activation of FPR2 by WKYMVm in human astrocytoma cells also results in Ca^2+^ influx and in the phosphorylation of inhibitory-κB kinase (IKK), which is prevented by pre-treatment of PTX and requires ERKs, PI3K and c-Src activation. Interestingly, cholesterol depletion from the plasma membrane abolishes the FPR2-stimulated IKK phosphorylation, denoting the important role of lipid raft integrity in the FPR2 to IKK signaling [130,131].

Chemokine receptors are subjected to heterologous desensitization by activation of formyl peptide receptors. Stimulation of human monocyte-differentiated immature dendritic cells (iDC) with WKYMVm results in a PKC-dependent phosphorylation of CCR5 and, in turn, in the downregulation of CCR5 from the cell surface and in a reduced cell response to the CCR5 ligands [132]. The same results are observed in human osteosarcoma cells, where the chemokine receptor, CXCR4, is desensitized upon stimulation with WKYMVm, thereby attenuating its biological functions [133]. In a mouse model, the administration of WKYMVm protects against death by enhanced bactericidal activity, by vital organ inflammation and by immune cell apoptosis. WKYMVm exerts these effects by enhancing the production of type 1 (IFN-γ and IL-12) and type 17 (IL-17 and TGF-β) cytokines and by inhibiting the production of pro-inflammatory cytokines, TNF-α, IL-1β and IL-6. The therapeutic, anti-inflammatory and bactericidal effects of WKYMVm is prevented by WRW4 and partly reversed in IFN-γ– and IL-17-deficient mice [134].

In FPR2-expressing RBL-2H3, cells the stimulation of FPR2 by WKYMVm induces ERKs phosphorylation and serine phosphorylation of STAT3 in a PTX-sensitive manner. Moreover, downstream of FPR2 stimulation, PLD activity is dramatically increased and n-butanol, a well-known phosphatidic acid acceptor, which completely inhibits WKYMVm-induced STAT3 serine phosphorylation [135] (Table 8).

The peptide, LESIFRSLLFRVM (MMK-1), was identified from a library screen in genetically engineered yeast cells designed to couple FPR2 activation to histidine prototrophy [136]. MMK-1 induces calcium mobilization in human cells transfected with FPR2 and is a potent chemotactic and calcium-mobilizing agonist for human monocytes and neutrophils [136]. Furthermore, MMK-1 shares the ability with other FPR2 agonists to induce NADPH oxidase-dependent superoxide generation in neutrophils [137]. FPRL1 inhibitory protein (FLIPr)-like is a chemoattractant-inhibiting protein derived from *Staphylococcus aureus*, which prevents neutrophil calcium mobilization induced by MMK-1 [138], whereas FLIPr inhibits calcium mobilization in neutrophils stimulated with MMK-1, WKYMVm, PrP^106–126^ and Aβ42 [139].

TIPMFVPESTSKLQKFTSWFM-amide (CGEN-855A) is a 21 amino acids peptide isolated by a computational platform designed to predict novel GPCR agonists cleaved from secreted proteins by convertase proteolysis [140]. It triggers calcium mobilization and an increase of the cell impedance index in cells expressing either FPR2 or FPR3, but does not affect monocyte secretion of cytokines. *In vivo*, CGEN-855A displays anti-inflammatory activity and protection against ischemia-reperfusion-mediated injury to the myocardium, which is accompanied by inhibition of PMN recruitment to the injured organ [140].

The MMHWAM peptide has been identified by screening a synthetic hexapeptide combinatorial library as a selective agonist of FPR2. In neutrophils and monocytes, MMHWAM promotes an increase of intracellular Ca^2 +^ via PLC activity, induces chemotaxis and stimulates superoxide anion production. The biological responses elicited by this peptide are prevented by PTX [141] (Table 9).

## 6. Ligands from Nonpeptide Library

Quinazoline is composed of fused benzene and pyrimidine rings. A quinazoline derivative (Quin-C1; 4-butoxy-*N*-[2-(4-methoxy-phenyl)-4oxo-1,4-dihydro-2*H*-quinazolin-3-yl]-benzamide) has been identified in a screening protocol based on FPR2-mediated reporter gene expression and calcium signaling as a highly selective agonist for FPR2 [142]. In neutrophils and in FPR2-expressing cells, Quin-C1 induces chemotaxis and secretion of β-glucuronidase. In rat basophilic cell lines expressing FPR2, it promotes calcium mobilization and stimulates ERKs phosphorylation. Quin-C1 does not induce superoxide generation in neutrophils and exhibits lower efficacy than WKYMVm in degranulation assays, suggesting that it selectively stimulates some FPR2-mediated functions [142]. Treatment with Quin-C1 significantly reduces, via FPR2, the expression of TNF-α, IL-1β, keratinocyte-derived chemokine, TGF-β1 and CXCL10 in a mouse model of bleomycin-induced lung injury, suggesting an anti-inflammatory role for this compound [143].

Pyrazolone, a five-membered-ring lactam, is a derivative of pyrazole with an additional keto group. Pyrazolone 24 and Pyrazolone 43 have been identified from a cell-based assay for high-throughput screening as selective agonists for FPR2 [144]. They exhibit anti-inflammatory properties and are able to stimulate calcium mobilization in FPR2-transfected cells.

Two aryl carboxylic acid hydrazide derivatives ([5-(3-bromophenyl)-2-furyl]-methylene-hydrazide and [5-(3-trifluoromethyl-phenyl)-2-furyl]-methylene-hydrazide) are selective FPR2 agonists [145]. These compounds induce PTX-sensitive production of TNFα in human and murine monocyte/macrophage cell lines and in primary macrophages, as well as mobilization of intracellular Ca^2+^, production of reactive oxygen species and chemotaxis in human and murine phagocytes.

Pyridazin is a heteroaromatic organic compound, also known as 1,2-diazine. Three pyridazin-3(2*H*)-one derivatives are agonists for FPR2 [146]. The compounds, *N*-(4-Bromophenyl)-2-[5-(3-methoxybenzyl)-3-methyl-6-oxo-6*H*-pyridazin-1-yl]-acetamide and *N*-(4-Bromophenyl)-2-[5-(4-methoxybenzyl)-3- methyl-6-oxo-6*H*-pyridazin-1-yl]-acetamide are mixed FPR1/FPR2 ligands, whereas the compound, *N*-(4-Bromophenyl)-2-[5-(4-methoxybenzyl)-3-methyl-6-oxo-6*H*-pyridazin-1-yl]-acetamide is a potent and specific FPR2 agonist. All these compounds activate intracellular calcium mobilization and chemotaxis in human neutrophils [146].

The screening of a chemolibrary of drug-like molecules allowed the identification of FPR nonpeptide agonists able to induce calcium mobilization in FPR-transfected RBL-2H3 cells. Seven compounds (AG-26 and AG-09/4 through AG-09/8) are specific for FPR2. Among these, compounds AG-09/3 and AG-09/4 have a common *N*-phenyl-2-(4-phenylpiperazin-1-yl)acetamide scaffold. Compounds AG-09/9, AG-09/10 and AG-22 are mixed FPR1/FPR2 agonists [84].

Thirty two ligands (agonists and antagonists) of unrelated GPCRs were screened for their ability to induce calcium mobilization in human neutrophils and HL-60 cells transfected with human FPR1, FPR2 or FPR3 [147]. The results demonstrate that two antagonists of gastrin-releasing peptide/neuromedin B receptors (BB1/BB2), PD168368 [(*S*)-a-methyl-a-[[[(4-nitrophenyl)amino] carbonyl]amino]-*N*-[[1-(2-pyridinyl)cyclohexyl]methyl]-1*H*-indole-3-propanamide] and PD176252 [(*S*)-*N*-[[1-(5-methoxy-2-pyridinyl)cyclohexyl]methyl]-a-methyl-a-[[-(4-nitrophenyl)amino]carbonyl] amino-1*H*-indole-3-propanamide], are potent mixed FPR1/FPR2 agonists. Cholecystokinin-1 receptor agonist A-71623 [Boc-Trp-Lys(ɛ-*N*-2-methylphenylaminocarbonyl)-Asp-(*N*-methyl)-Phe-NH_2_] is also a mixed FPR1/FPR2 agonist. These ligands are potent chemoattractants and activate reactive oxygen species generation in human neutrophils [147] (Table 10).

### Allergens

Allergen-induced airway inflammation may lead to allergic asthma, a chronic inflammatory disease of the respiratory system. The mammalian airway epithelia constitutively express uteroglobin (UG) or Clara cell 10 kDa protein (CC10), a steroid-inducible secreted protein with potent anti-inflammatory and anti-chemotactic properties. UG binds to FPR2 with high specificity and prevents allergen-induced expression of the suppressor of cytokine signalling-3 (SOCS-3) in the lungs, which regulates the initiation and maintenance of T_H_2-mediated allergic airway inflammatory responses. STAT-1 activation plays a critical role in SOCS-3 gene expression, and UG, acting on FPR2 as an antagonist, inhibits allergen-induced STAT-1 mRNA expression. These observations suggest that UG suppresses SOCS-3 gene expression by downregulating allergen-induced expression and phosphorylation/activation of STAT-1 and, in turn, the differentiation of T_H_2 cells [148]. House dust mite (HDM) and birch pollen extracts activate chemotaxis and degranulation in human eosinophils, which express FPR1 and FPR2. Inhibition and desensitization of these receptors renders eosinophils anergic to activation by allergens [149]. HDM evokes calcium fluxes in HL-60 cells transfected with FPR1 or FPR2, and in neutrophils, PTX pre-treatment and FPR antagonists prevent HDM-mediated migration of these cells. Furthermore, eosinophils preincubated with inhibitors of p38MAPK, ERK1/2 or protein kinase C show attenuated responsiveness to the aeroallergens, suggesting that allergens and FPR2 agonists use similar transduction molecules to activate eosinophils [149].

## 7. Conclusions

FPR2 shows complex functional properties, partly due to its high promiscuity, but also due to the fact that its activation can stimulate several signal transduction pathways, depending on the ligand, its concentration and the cell type involved. Several endogenous FPR2 agonists also present in biological samples have been identified. They include lipids, such as LXA4 and resolvins, and proteins, such as SAA, Aβ42, HN, PrP^106–126^, uPAR, LL-37, chemokine variants, the neuropeptides, VIP and PACAP27, ANXA1 and derived peptides and mitochondrial peptides. The study of endogenous ligands for FPR2 provides evidence of pro-inflammatory and anti-inflammatory downstream responses, even though the characterization of the pharmacological properties of synthetic agonists suggests a prominent anti-inflammatory role in the contest of host defense. On the other hand, the use of FPR2 by Aβ42 and PrP^106–126^ suggests that this receptor may play a crucial role in pro-inflammatory aspects of AD and prion diseases. FPR2 responds to synthetic ligands, and the newly identified agonists do not share sequence homology, which suggests that this receptor can be activated by a wide variety of unrelated ligands that can also be generated during pathological conditions. FPR2 plays important roles in various diseases and is strongly implicated in cancer. In fact, it is involved in motility, growth and angiogenesis of human tumors, triggering specific antitumor host immune responses. The activation of the PI3K-Akt/PKB pathway mediated by FPR2 supports tumor cell survival and proliferation in several cell types. NADPH oxidase activation, the mitogen-activated protein kinases, ERK1/2, JNK and p38MAPK, are also activated in several cells types stimulated with different FPR2 agonists. Activation of the MEK/ERK pathway plays a key role in cell proliferation, in protection against cell death, in the regulation of NADPH oxidase and in the transcriptional factor complex activation. JNK, p38MAPK and JAK/STAT signaling seem to be implicated in increasing angiogenesis and in the malignant behavior observed in several human cancer cell lines.

Further studies are required to define the complete dissection of the intracellular signaling pathways triggered by different agonists of FPR2 in physiological and pathological conditions.

## Figures and Tables

**Table 1:** Intracellular signaling cascades triggered by microbe-derived peptides.

Ligand	Origin	Selectivity	Cells	Effects	Ref.	Potency
Hp(2–20)	*H. pylori*	FPR2, FPR3 FPR2	Mon.; Lymph. MKN-28, AGS	O_2_^−.^ generation; apoptosis; chemotaxis; proliferation; VEGF secretion; ERKs, Akt and STAT3 activation	[6,9]	pEC_50_ = 6.52
F peptide	HIV-1	FPR2	Mon.; Neutr.	chemotaxis; Ca^2+^ mobilization; desensitization CCR5 and CXCR4	[7,10]	pEC_50_ = 5.00
V3 peptide	HIV-1	FPR2	Mon.; Neutr.	chemotaxis; Ca^2+^ mobilization; desensitization CCR5	[11]	pEC_50_ = 5.82
T21/DP107	HIV-1	FPR2	Mon.; Neutr.	chemotaxis; Ca^2+^ mobilization;	[12]	pEC_50_ = 6.30
N36 peptide	HIV-1	FPR2	Mon.; Neutr.	chemotaxis; Ca^2+^ mobilization; desensitization chemokine receptors; increased expression of TRAIL; NFκB activation; apoptosis	[13,14]	pEC_50_ = 5.00
PSMs	Peptide toxins	FPR2	Neutrophils	Ca^++^ mobilization; chemotaxis; IL-8 release; NADPH oxidase activation	[15,16]	pEC_50_ = 8.67

FPR, formyl-peptide receptor; Mon., monocytes; Lymph., lymphocytes; Neutr., neutrophils; VEGF, vascular endothelial growth factor; ERK, extracellular signal-regulated kinase; Akt, protein kinase B; STAT3, signal transducer and activator of transcription 3; TRAIL, tumor necrosis factor-related apoptosis-inducing ligand; NADPH, nicotinamide adenine dinucleotide phosphate; pEC_50_, negative logarithm of the EC_50_.

**Table 2:** Intracellular signaling cascades triggered by mitochondrial peptides.

Ligands	Origin	Selectivity	Cells	Effects	Ref.	Potency
f-MLKLIV	Mitochondria	FPR1, FPR2	FPR-transfected HL60	chemotaxis; Ca^2+^ mobilization; ERKs activation	[8]	pEC_50_ = 7.92, 7.26
f-MMYALF	Mitochondria	FPR2 > FPR1	FPR-transfected HL60	chemotaxis; O_2_^−.^ generation	[8]	pEC_50_ = 7.82, 7.92
MCT-2	Mitochondria	FPR2	Neutrophils; Granulocytes	chemotaxis; Ca^2+^ mobilization; ERKs activation β-hexosaminidase release	[18]	EC_50_ = 240 nM

pEC_50_ = negative logarithm of the EC_50_.

**Table 3:** Intracellular signaling cascades triggered by amyloidogenic peptides and proteins.

Ligand	Origin	Selectivity	Cells	Effects	Ref.	Potency
SAA	Acute-phase protein	FPR2	Mon.; Neutr.; Lymph.	chemotaxis; Ca^2+^ mobilization; production of metalloproteases and cytokines; expression of cytokine receptors; ERKs, JNK and p38MAPK activation; COX2 and NF-κB induction; IL-8, IL-10 and TNF-α release; MMP-9 upregulation	[3,21]	pEC_50_ = 7.35
			FLS	proliferation; anti-apoptosis; ERKs and Akt activation; expression of MMP-1 and -3; IL-6 production	[25–27]	
			Endothelial cells	production of PTX3	[30]	
Aβ42	Amyloid precursor		Mon.; RBL; FPR2/HEK293	chemotaxis; Ca^2+^ mobilization; O_2_^−.^ generation	[4,36]	pEC_50_ = 7.00
			Glial cells	ERKs phosphorylation; PI3K/Akt pathway; PLD activation; FPR1/FPR2/MARCO physical and functional interaction	[35,37]	EC_50_ = 5 μM
HN	Neuroprotective peptide		Mononuclear phagocytes	Chemotaxis; Ca^2+^ mobilization; anti-apoptosis; ERKs phosphorylation	[38]	pEC_50_ = 8.46
PrP_106–126_	Prion protein		Glial cells	protein tyrosine phosphorylation; IL-6 and TNF-α increase; chemotaxis; Ca^2+^ mobilization	[42]	pEC_50_ = 4.60
			Ana-1	Chemotaxis	[43]	

Aβ42, β-amyloid peptide 42; HN, humanin; FLS, fibroblast-like synoviocytes; JNK, c-Jun N-terminal kinase; COX2, cyclooxygenase-2; MMP-9, matrix-metalloproteinase-9; PTX3, pentraxin 3; PLD, phospholipase D; MARCO, macrophage receptor with collagenous structure; Mon., Monocytes; Neutr., Neutrophils; Lymph., Lymphocytes; pEC_50_, negative logarithm of the EC_50_.

**Table 4:** Intracellular signaling cascades triggered by peptides associated with inflammatory and anti-inflammatory responses.

Ligand	Origin	Selectivity	Cells	Effects	Ref.	Potency
D2D3_88–274_	uPAR	FPR2	THP-1; Mon.	chemotaxis; decreased chemokine-induced integrin-dependent cell adhesion	[5,48]	pEC_50_ = 7.08
uPAR_84–95_	uPAR	FPR2, FPR3	Basophils	chemotaxis	[5]	Kd = 82.6
LL-37	Cathelicidin	FPR2	FPR2/HEK293; Lymph.; Mon.	chemotaxis; Ca^2+^ mobilization	[51]	pEC_50_ = 6.00
			Neutrophils	Anti-apoptosis; Bcl-xL expression; inhibition of caspase 3 and of SAA-induced IL-8 production; inhibition of SAA-induced ERKs and p38MAPK activity; LTB4 production; cPLA2 phosphorylation	[52,53,59,60]	
			Endothelial cells	vessel growth; Ca^2+^ mobilization; NF-κB nuclear translocation; PKC activation; O_2_^−.^ generation; ERKs phosphorylation	[54]	
			NCI-H292	proliferation; migration; wound healing	[55]	
			MSCs	inhibition of tumor growth; ERKs phosphorylation	[56]	
			Ovarian cancer cells	MAPK and JAK/STAT signaling; expression of angiopoietin-like 3, C5, collagen type XVIII, EGF, FGF1, FPR2, LL-37, MMP-2, uPA	[57]	
			Hepatocarcinoma	M-CSF and MCP-1 expression; ROS-MAPK-NFκB signaling	[58]	
			IMR9	O_2_^−.^ generation; p47^phox^ and ERKs phosphorylation	[61]	
sCKβ8-1	Chemokine	FPR2	PMN, FPR2/CHO-K1	chemotaxis; Ca^2+^ mobilization	[62]	pEC_50_ = 9.00
SHAAGtide	CCL23	FPR2	Mon.; Neutr.	chemotaxis; Ca^2+^ mobilization	[63]	pEC_50_ = 7.72
VIP	Pleiotropic peptide	VPAC1, FPR2	Monocytes	pro-inflammatory; PI3K/ERK activation; CD11b upregulation	[67]	
PACAP27	Neuropeptide	FPR2	Neutrophils	chemotaxis; Ca^2+^ mobilization; CD11b upregulation; ERKs, Akt, p38MAPK phosphorylation	[69]	EC_50_ = 0.33 μM

uPAR, urokinase-type plasminogen activator receptor; PACAP, pituitary adenylate cyclase-activating polypeptide; VCAP1, VIP/pituitary adenylate cyclase-activating protein; MSC, mesenchymal stromal cell; PMN, human neutrophil; LTB4, leukotriene B4; PKC, protein kinase C; C5, complement 5; EGF, epidermal growth factor; FGF1, fibroblast growth factor 1; MMP-2, metalloproteinase-2; M-CSF, macrophage colony-stimulating factor; MCP-1, monocyte chemoattractant protein-1; Mon., Monocytes; Lymph., Lymphocytes; Neutr., Neutrophils; pEC_50_, negative logarithm of the EC_50_.

**Table 5:** Intracellular signaling cascades triggered by annexin A1 and derived peptides.

Ligand	Origin	Selectivity	Cells	Effects	Ref.	Potency
antiflammin-2	ANXA1	FPR2	FPR2/HEK293	ERKs phosphorylation	[76]	EC_50_ = 1.2 μM
Ac2-26	ANXA1	FPR1 > FPR2	PMN; FPR1/HE293; FPR2/HEK293	ERKs phosphorylation	[73]	pEC_50_ = 6.05 EC_50_ = 25 μM
		FPR2	Synovial fibroblasts	MMP-1 secretion	[78]	
		FPR1 > FPR2	MDA-MB-231	Cell proliferation	[81]	
ANXA1	ANXA1	FPR2	PMN; FPR2/HEK293	ERKs phosphorylation	[73]	EC_50_ = 0.15 μM EC_50_ = 25 μM
			Synovial fibroblasts	MMP-1 secretion	[78]	
			MCF-7	PI3K/Akt/p70S6K pathway; cyclin D increase	[79]	
			SKCO-15	Cell invasion	[80]	
			Lung fibroblasts	TNFα-induced cell proliferation; inflammatory responses; activation of ERK and NF-κB pathways	[82]	

ANXA1, annexin A1, pEC_50_, negative logarithm of the EC_50_.

**Table 6:** Intracellular signaling cascades triggered by other endogenous peptides.

Ligand	Origin	Selectivity	Cells	Effects	Ref.	Potency
TA	Antimicrobial peptide	FPR2	Mon.; Neutr.; Macroph.	chemotaxis; Ca^2+^ mobilization; ERKs activation	[85]	pEC_50_ = 6.60
I4S10-C	Antimicrobial peptide	FPR2	FPR2/HEK293	Cell migration	[85]	EC_50_ = 5 μM
I4G10-C	Antimicrobial peptide	FPR2	FPR2/HEK293	Cell migration	[85]	EC_50_ = 0.5 μM
Rana-6	Antimicrobial peptide	FPR2	FPR2/HEK293	Cell migration	[85]	EC_50_ = 5 μM
L37pA	apoA-I	FPR2	Mon.; FPR2/HEK293	chemotaxis; Ca^2+^ mobilization; anti-inflammatory	[86]	EC_50_ = 112 nM

Mon, Monocytes; Neutr., Neutrophils; Macroph., Macrophages; pEC_50_, negative logarithm of the EC_50_.

**Table 7:** Intracellular signaling cascades triggered by endogenous nonpeptide ligands.

Ligand	Origin	Selectivity	Cells	Effects	Ref.	Potency
LXA4	Eicosanoids	FPR2	Epith. and Endothel. cells; Neutrophils	NO production; inhibition of neutrophil infiltration and transmigration	[93–95]	p*K*d = 8.77 EC_50_ = 50 nM
			Neutrophils	chemotaxis; Ca^2+^ mobilization; PKC-dependent PLD activation	[96]	
			Renal mesangial cells	inhibition of LTD4- and LXA4-induced cell proliferation and PI3K activity; ERKs and p38MAPK phosphorylation; inhibition of PDGF-Rβ and EGF-R; p21^cip1^ and p27^kip1^ modulation; inhibition of PDGF-induced increase of CDK2/cyclin E complex; block of G1-S progression	[97,98]	
			HLF	inhibition of CTGF-induced cell proliferation, of ERKs, PI3K and Akt phosphorylation, of cyclin D1 expression and of STAT3 DNA-binding activity; p27^kip1^ modulation	[99]	
			MCF-7; MDA-MB-231	increase in cyclin D1; Akt and p79S6K phosphorylation	[81]	
			Synovial fibroblasts	inhibition of IL-1β-induced IL-6, IL-8 and MMP-3 synthesis of FPR2 expression; downregulation of IL-1β-induced AP1 and NF-κB DNA binding activity	[100,101]	
			Dendritic cells	induction of SOCS-2	[106]	
Rv	Lipid mediator	GPCR-32, FPR2	Salivary cells	cell migration; polarity; inhibition of TNF-α-induced cytoskeletal disruption; modulation of PI3K/Akt pathway	[108]	-
D1		FPR2	Acute lung injury	decrement of IL-1β, IL-6, TNF-α and of NF-κB p65 translocation	[109]	
		FPR2	Inflamed adipose tissue	secretion of adiponectin; decreased pro-inflammatory adipokine production	[110]	

LXA4, lipoxin A4; GPCR, G-protein coupled receptor; HLF, human lung fibroblast; PDGF, platelet-derived growth factor; CTGF, connective tissue growth factor; Epith. and Endothel. cells, Epithelial and Endothelial cells; p*K*d, negative logarithm of *K*d.

**Table 8:** Intracellular signaling cascades triggered by WKYMVm.

Ligand	Origin	Selectivity	Cells	Effects	Ref.	Potency
WKYMVm	Peptide library	FPR2	Neutrophils	increase in Ca^++^ concentration; NADPH oxidase activation; cPLA2-mediated arachidonic acid release; increase of LTB4 production	[115,120]	pEC_50_ = 8.70
			Monocytes	chemotaxis; p125FAK, Pyk, MEK, ERKs, Akt and RhoA phosphorylation; NADPH oxidase activation; PKC and PLD activation	[118,121]	EC_50_ = 50 nM
			Eosinophils	ERKs phosphorylation; NADPH oxidase activation; PI3K/ERK pathway	[122]	
			U937	ERKs phosphorylation; G_0_/PI3K/Ras/Raf-1 pathway; cPLA and PLD activation; LPA formation; Ca^++^ influx	[123,124]	pEC_50_ = 10.13
			NK	chemotaxis in IL-2-activated NK cells; ERKs, p38MAPK and JNK activation	[125]	EC_50_ = 9.2 nM
			IMR90	ERKs activation; p47^phox^ translocation; NADPH oxidase, PKCα and PKCδ activation	[126,127]	*K*d = 155,99 nM
			CaLu-6	EGFR transactivation, p47^phox^ phosphorylation, NADPH oxidase activation; c-Src activation; STAT3 pathway; cell growth	[128]	-
			U87	ERKs, p38MAPK and JNK activation; c-Src and PLCβ activation; GFAP and IL-1α upregulation; IKK phosphorylation; PI3K activation; Ca^++^ influx	[129–131]	EC_50_ = 50–100 nM
			iDC	downregulation of CCR5; PKC activation	[132]	
			Osteosarcoma	downregulation of CXCR4	[133]	
			Mouse model	Anti-apoptosis; enhanced production of IFN-γ, IL-12, IL-17 and TGF-β; reduced production of TNF-α, IL-1β and IL-6	[134]	
			FPR2/RBL-2H3	ERKs phosphorylation; STAT3 serine phosphorylation; PLD activation	[135]	

LPA, lysophosphatidic acid; IKK, inhibitory-κB kinase; pEC_50_, negative logarithm of the EC_50_.

**Table 9:** Intracellular signaling cascades triggered by other ligands from peptide library.

Ligand	Origin	Selectivity	Cells	Effects	Ref.	Potency
MMK-1	Peptide library	FPR2	Neutrophils; Monocytes	Ca^++^ mobilization; chemotaxis; NADPH oxidase activation	[136,137]	pEC_50_ = 8.70
CGEN-855A	Peptide library	FPR2, FPR3	FPR2/3-expressing cells	Ca^++^ mobilization; increase of cell impedance index; anti-inflammatory	[140]	IC_50_ = 189 nM Ki = 54.1 nM
MMHWAM	Peptide library	FPR2	Neutrophils; Monocytes	Ca^++^ mobilization; chemotaxis; PLC activation; NADPH oxidase activation	[141]	-

pEC_50_, negative logarithm of the EC_50_.

**Table 10:** Intracellular signaling cascades triggered by ligands from nonpeptide library.

Ligand	Origin	Selectivity	Cells	Effects	Ref.	Potency
Quin-C1	Combinatorial library	FPR2	Neutrophils; FPR2-expressing cells	chemotaxis; β-glucuronidase secretion	[142]	pEC_50_ = 5.72
			FPR2/RBL	Ca^2+^ mobilization; ERKs activation	[142]	
			Mouse model	anti-inflammatory; reduction of the expression of TNF-α, IL-1β, keratinocyte-derived chemokine, TGF-β1 and CXCL10	[143]	
Pyrazolone 24/43	Combinatorial library	FPR2	FPR2-transfected cells	anti-inflammatory; Ca^2+^ mobilization	[144]	pIC_50_ = 7.36
Aryl carboxylic acid hydrazide derivatives	Chemolibrary of drug-like molecules	FPR2	Monocytes; Macrophages; Phagocytes	TNFα production; Ca^2+^ mobilization; reactive oxygen species production; chemotaxis	[145]	EC_50_ = 2 μM
Pyridazin-derivatives	Ligand-based drug design approach	FPR1/FPR2	Neutrophils	Ca^2+^ mobilization; chemotaxis	[146]	EC_50_ = 13.1 μM
AG-26, AG-09/4-AG09/8	Chemolibrary of drug-like molecules	FPR2	FPR2-transfected RBL-2H3	Ca^2+^ mobilization	[84]	EC_50_ = 0.5 μM, 0.3–12.6 μM
PD168368; PD176252; A-716223	Screening of known GPCR ligands	FPR1/FPR2	FPRs-transfected HL-60; Neutrophils	Ca^2+^ mobilization; reactive oxygen species production	[147]	EC_50_ = 0.5 μM, 0.9 μM, 18.3 μM

pEC_50_, negative logarithm of the EC_50_; pIC_50_, negative logarithm of the IC_50_.
